# The Roles of Transient Receptor Potential Ion Channels in Pathologies of Glaucoma

**DOI:** 10.3389/fphys.2022.806786

**Published:** 2022-02-03

**Authors:** Lin Ma, Xin Liu, Qing Liu, Sen Jin, Heng Chang, Haixia Liu

**Affiliations:** ^1^Department of Ophthalmology, Tongji Hospital, Tongji Medical College, Huazhong University of Science and Technology, Wuhan, China; ^2^Shenzhen Key Laboratory of Viral Vectors for Biomedicine, Chinese Academy of Sciences, Shenzhen Institute of Advanced Technology, Shenzhen-Hong Kong Institute of Brain Science-Shenzhen Fundamental Research Institutions, The Brain Cognition and Brain Disease Institute, Shenzhen, China; ^3^University of Chinese Academy of Sciences, Beijing, China

**Keywords:** glaucoma, transient receptor ion potential channels, intraocular pressure, retinal ganglion cells, membrane protein

## Abstract

Transient receptor ion potential (TRP) channels are a cluster of non-selective cation channels present on cell membranes. They are important mediators of sensory signals to regulate cellular functions and signaling pathways. Alterations and dysfunction of these channels could disrupt physiological processes, thus leading to a broad array of disorders, such as cardiovascular, renal and nervous system diseases. These effects position them as potential targets for drug design and treatment. Because TRP channels can mediate processes such as mechanical conduction, osmotic pressure, and oxidative stress, they have been studied in the context of glaucoma. Glaucoma is an irreversible blinding eye disease caused by an intermittent or sustained increase in intraocular pressure (IOP), which results in the apoptosis of retinal ganglion cells (RGCs), optic nerve atrophy and eventually visual field defects. An increasing number of studies have documented that various TRP subfamilies are abundantly expressed in ocular structures, including the cornea, lens, ciliary body (CB), trabecular meshwork (TM) and retina. In alignment with these findings, there is also mounting evidence supporting the potential role of the TRP family in glaucoma progression. Therefore, it is of great interest and clinical significance to gain an increased understanding of these channels, which in turn could shed more light on the identification of new therapeutic targets for glaucoma. Moreover, this role is not understood completely to date, and whether the activation of TRP channels contributes to glaucoma, or instead aggravates progression, needs to be explored. In this manuscript, we aim to provide a comprehensive overview of recent research on TRP channels in glaucoma and to suggest novel targets for future therapeutic interventions in glaucoma.

## Introduction

Glaucoma, the age-related neuropathy, is the leading cause of irreversible global vision loss (Tham et al., 2014). Multiple risk factors have been identified in glaucoma pathophysiology, including but not limited to genetic abnormalities, intraocular pressure (IOP), race, age, and gender. IOP is widely accepted as the most important yet also the only modifiable risk factor (Quigley, 2011). The elevated mechanical pressure on the optic nerve damages the optic nerve head and causes consequential visual impairment if left untreated. The modern goals of glaucoma management are to lower IOP and avoid optic nerve damage, with minimal side effects (Tham et al., 2014). Mechanotransduction channels have been studied extensively in glaucoma (Hirt and Liton, 2017; Yarishkin et al., 2021). Recent extensive research regarding transient receptor ion potential (TRP) channels in glaucoma pathogenesis has also raised scientists’ interest in targeting these channels for novel therapeutic development.

Transient receptor ion potential channels, which were first discovered in visual conduction research of Drosophila, are a group of protein superfamily structures located mostly on the plasma membrane (Montell and Rubin, 1989). To date, twenty-eight TRP channel genes have been identified in mammals, which can be divided into six subfamilies based on the homology of the gene sequence, namely TRPV (Vanilloid), TRPA (Ankyrin), TRPC (Canonical), transient receptor potential melastatin receptor (TRPM) (Melastatin), TRPN (NOMPC), TRPP (Polycystin), and TRPML (Mucolipin) (Montell, 2005; Nilius and Szallasi, 2014). TRP channels are composed of six transmembrane domains (S1-S6), in which the pore structure between the S5 and S6 segments allows permeation of Na^+^ and Ca^2+^ ions and trace metal ions. Permeability generates action potentials in the cell membrane and transmits signals to the spinal cord and central nervous system (Nilius and Owsianik, 2011). The distinguishing characteristics between TRP channels have been reported in the N- and C-terminal cytosolic domains, which contain unique regulatory motifs for each corresponding subfamily for each subfamily (Gaudet, 2008). In addition, TRP channels can mediate a variety of stimuli, including sensory stimulation, osmolarity changes, mechanical stimuli and chemical stimuli (Kung, 2005; Liedtke and Kim, 2005; Christensen and Corey, 2007; Sharif-Naeini et al., 2008). Moreover, they are also involved in the oxidative stress response (Yamamoto and Shimizu, 2016). During the onset of glaucoma, the elevation of IOP causes retinal ischemia and hypoxia, along with oxidative stress accumulation, all of which contribute to optic nerve degeneration and deterioration of retinal ganglion cells (RGCs) (Kimura et al., 2017). Evidence showed that TRPs of eyes have critical roles in modulating IOP and retinal function. For example, they are involved in the secretion of aqueous humor (AH), and in cytoskeleton remodeling of the trabecular meshwork (TM) to control IOP. A thorough understanding of the relationship between TRPs and glaucoma is of great clinical importance for the study of glaucoma pathogenesis, glaucoma treatment and the development of new drug therapeutic targets. In this review, we aim to clarify the functional involvement of TRP channels in mediating glaucoma progression through modulating retinal and TM function, which may be useful in the exploitation of novel targets for future therapeutic intervention in glaucoma.

## Involvement of Transient Receptor Ion Potential Channels in the Development of Glaucoma

### Transient Receptor Potential Vanilloid 1 Channel

#### Expression of Transient Receptor Potential Vanilloid 1 Channel in Ocular Structures Associated With the Pathogenesis of Glaucoma

In the eyeball, the expression of the transient receptor potential vanilloid receptor 1 (TRPV1) channel is confined to a subset of RGCs (Gilliam and Wensel, 2011), with an apparent increase at the transition from the central to the mid-peripheral retina. In addition, the *Trpv1* gene continues to be regularly transcribed and translated in the adult retina (Jo et al., 2017). In addition, this channel is also observed in glial cells of the retina, such as astrocytes and microglia (Sappington et al., 2009).

#### Function of the Transient Receptor Potential Vanilloid 1 Channel in Glaucoma

Alzheimer’s disease (Jayant et al., 2016; Balleza-Tapia et al., 2018), Parkinson’s disease (Marinelli et al., 2003) and Huntington’s disease (Lastres-Becker et al., 2003) are all central neurodegenerative disorders, and TRPV1 protein has been proven to have potential therapeutic effects on those diseases (Du et al., 2020; Duitama et al., 2020). The optic nerve is an extension of the central nervous system (CNS), and the axonal degeneration of the optic nerve is essentially similar to central neurodegenerative diseases (Gupta et al., 2006). Therefore, an increasing number of people pay attention to the functional properties of TRPV1 in the optic nerve during the progression of glaucoma. TRPV1 channel activation is often accompanied by strong calcium influx (Agopyan et al., 2004); however, TRPV1 activation results in Ca^2+^ transients that can lead to cell death if they reach too high levels and are sustained (Reilly et al., 2005). Initially, Sappington found that inhibition of the channel in isolated RGCs exposed to elevated hydrostatic pressure can reduce increased intracellular Ca^2+^ and RGC apoptosis, suggesting that TRPV1 antagonism could become a novel target for therapy (Sappington et al., 2009). Later, Ward et al. (2014) pointed out that either TRPV1 knockout or pharmacological inhibition of the channel could accelerate optic nerve axonopathy with elevated IOP and increase the membrane depolarization indispensable for RGCs to generate action potentials, therefore TRPV1 may boost excitatory activity as a means of promoting RGCs survival in glaucoma. Subsequent research further indicated that TRPV1 mRNA levels in RGCs increased slightly with elevated IOP (Weitlauf et al., 2014), which is consistent with the phenomenon observed in DBA/2J mice in which TRPV1 localization rises transiently in RGCs (Sappington et al., 2009). Further studies reported that the channel could increase excitability changes as well. TRPV1 may promote RGC survival through transient enhancement of local excitation and axonal activity in response to ocular stress (Weitlauf et al., 2014).

In addition, the presence of the TRPV1 channel is also observed in astrocytes and microglia of the retina, which are glial cells and have a trophic role in neurons (Sappington et al., 2009). In addition, they also participate in the process of information transmission between neurons (Kettenmann et al., 2011; Prasanna et al., 2011). When exposed to elevated pressure *in vitro*, the retinal microglia are activated, and the secretion of interleukin 6 (IL-6) subsequently increases. IL-6 is reported to have a protective effect on RGCs exposed to high IOP (Sappington and Calkins, 2006; Sappington et al., 2006). Later, Sappington pointed out that Ca^2+^ is a strong modulator of pressure-induced IL-6 release and that TRPV1 in microglia can modulate the stimulus-induced production of IL-6, suggesting that TRPV1 may contribute to this process (Sappington and Calkins, 2008). To better understand the early stages of the acceleration of optic nerve degeneration, Nolan et al. showed that genetic ablation of the TRPV1 channel increased voltage-gated sodium channel subunit 1.6 (NaV1.6) and excitability of RGC axons in the retina following short-term elevation in IOP, suggesting that TRPV1 may redistribute the expression of NaV in response to pressures to normalize excitability to available metabolic resources (McGrady et al., 2020). Taken together, whether activation of TRPV1 is protective or toxic to RGCs might depend greatly on the expression levels because of this channel’s high Ca^2+^ conductance, the role of TRPV1 in glaucoma needs more studies to be better understood.

### Transient Receptor Potential Vanilloid 4 Channel

#### Expression of Transient Receptor Potential Vanilloid 4 Channel in Ocular Structures Associated With the Pathogenesis of Glaucoma

In the eyeball, the transient receptor potential vanilloid receptor 4 (TRPV4) has been studied extensively in the TRP family concerning glaucoma. Functional expression of this channel has been identified in the ciliary body (CB) (Jo et al., 2016), TM (Ryskamp et al., 2016; Yarishkin et al., 2021) and retina (Ryskamp et al., 2011). Although the CB is comprised of non-pigmented epithelial cells (NPE), pigmented epithelial cells (PE) and ciliary muscle (Coca-Prados and Escribano, 2007), the TRPV4 channel is expressed selectively in NPE responsible for sensing osmotic alteration and secreting AH, which is important to nourish the avascular structures of the eye and to maintain the IOP within physiological values (Jo et al., 2016). In addition, TRPV4 is enriched in the primary cilia of TM cells, RGCs, optic nerve heads and Müller glial cells (Jo et al., 2015).

#### Function of the Transient Receptor Potential Vanilloid 4 Channel in Glaucoma

The TRPV4 protein has been studied as an osmotic pressure sensor to act as a regulator of paracellular permeability in a variety of epithelial tissues (Harteneck and Reiter, 2007; Loukin et al., 2010; Mamenko et al., 2015; Narita et al., 2015), and osmotic regulation impairment can be observed in TRPV4 null mice (Liedtke and Friedman, 2003). Altered osmotic gradients influence the process of ciliary fluid ultrafiltration and water secretion (Coca-Prados and Escribano, 2007). Furthermore, Andrew et al. found that the hypotonic effect produced by NPE cells swelling can directly trigger TRPV4-mediated Ca^2+^ inward flow. Additionally, they found that the TRPV4 channel could be indirectly activated by the PLA2 pathway’s release of arachidonic acid, eventually mediating large increases in AH formation (Jo et al., 2016). These shreds of evidence provide new insight into the mechanism of AH secretion and IOP modulation. Another study has shown that the TRPV4 selective agonist GSK101 could cause a concentration-dependent increase in the extracellular level of melatonin in human NPE cells (Alkozi and Pintor, 2015). Melatonin can reduce IOP through melatonin receptors present in this tissue by reducing rises in chloride production from the ciliary epithelium (Alarma-Estrany et al., 2009, 2011; Ismail and Mowafi, 2009; Martínez-Águila et al., 2013; Huete-Toral et al., 2015). The ciliary is responsible for the secretion of AH; however, the TM could filter AH through the conventional outflow pathway in humans to modulate the drainage of AH. The outflow channel is indispensable to maintain the balance of AH circulation, lesion occurred in the outflow system will cause increased outflow resistance and thus result in rised IOP (Weinreb et al., 2014). Previous studies reported conflicting results regarding the role of TRPV4 in TM on IOP regulation. In mice treated systemically and instillation of TRPV4 agonist GSK101, the IOP decreased in C57BL/6J mice owing to the increased outflow facility of TM (Jo et al., 2016; Patel et al., 2021; Uchida et al., 2021). Compared to TRPV4^+/+^ mice, TRPV4^–/–^ animals exhibited increased IOP and shortened primary cilia of TM. The normal primary cilia were identified as the critical regulator of mechanotransduction in the kidney epithelium and the lining of the ventricles, sensing changes in urine and cerebrospinal fluid, respectively. TRPV4 is possibly activated by increases in hydrostatic pressure or mechanical deformation induced by rises in IOP. These tissues located in the TM can also act as pressure-sensing organelles in mechanical transduction processes. TRPV4 is dependent on OCRL (an inositol polyphosphate 5-phosphatase) localization to primary cilia for proper localization and function. Under normal circumstances, TRPV4 interaction with OCRL may cause enhanced endothelial nitric oxide synthase, which has been documented to lower IOP by increasing pressure-dependent drainage (Stamer et al., 2011; Luo et al., 2014; Patel et al., 2021). However, in TRPV4^–/–^ mice, calcium signaling and the cilia growth process are impaired and can not respond well to mechanical stimuli, eventually resulting in diminished TM function and increased IOP (Stamer et al., 2011; Luo et al., 2014; Uchida et al., 2021). On the other hand, another study by Ryskamp et al. (2016) demonstrated that TRPV4 antagonists reduce the elevation of IOP in chronic hypertensive eyes. Considering that the methods of administration, conditions and animal age were different among these studies, there is currently no consensus on the regulation of TM *via* the TRPV4 channel with respect to IOP.

Transient Receptor Potential Vanilloid 4 is also involved in the apoptosis process of RGCs. GSK101 and 4α-PDD (TRPV4 agonists) also cause Ca^2+^ influx in RGCs so that sustained TRPV4 activation can compromise their survival, eventually resulting in dose-dependent apoptosis of RGCs (Ryskamp et al., 2011). Additionally, Müller cells are the primary type of retinal glial cells, which can support and regulate extracellular fluid within the retina (Reichenbach and Bringmann, 2013). The TRPV4 channel can interact with AQP4 (aquaporin 4) in Müller cells to regulate fluid exchange and calcium homeostasis (Jo et al., 2015). In fact, TRPV4 has been considered a potential therapeutic target to treat glaucoma because its inhibition has been found to improve the survival of RGCs (Taylor et al., 2017)Taken together, these studies highlight TRPV4 as a potential target in treating glaucoma.

### Transient Receptor Potential Family Member Ankyrin Channel

#### Expression of Transient Receptor Potential Family Member Ankyrin Channels in Ocular Structures Associated With the Pathogenesis of Glaucoma

In mammals, transient receptor potential family member ankyrin 1 (TRPA1) is concentrated and expressed in the endings of small- and medium-diameter primary sensory nerve fibers (such as spinal dorsal root ganglion, trigeminal ganglion, etc.) (Nagata et al., 2005). Its main physiological function is to sense cold, noxious stimuli such as pain and mechanical stress (Talavera et al., 2020). The channel plays an important role in pathological processes such as oxidative stress, blood vessel expansion and inflammation as well (Trevisan et al., 2014). To date, this protein has been found to be expressed in the trigeminal nerve endings in the anterior chamber (AC) of the rats in the eyeball and in various retinal cells such as ganglion cells, Müller cells and photoreceptor cells in mice (de Araujo et al., 2020).

#### Function of the Transient Receptor Potential Family Member Ankyrin 1 Channel in Glaucoma

The TRPA1 channel may be a sensor for detecting changes of the pressure in the AC. Initially, we used patch clamp technology and characterized the individual effects of allyl isothiocyanate (AITC) and HC-030031, TRPA1 specific agonists and antagonists, on changes in its channel behavior. These responses coupled with the knowledge that TRPA1 channels are mechanosensitive prompted suggestions that they may act as baroceptor detectors of changes in AC pressure. This function is like that of TRPV4, which transmits nerve impulses to the CNS for IOP neuromodulation (Meng et al., 2015). Subsequently, we also discovered with Ca^2+^ fluorescence imaging technology that bimatoprost (prostaglandin analog) is a TRPA1 agonist, which may activate trigeminal nerve endings distributed throughout the AC. Such an effect increases the mechanical sensitivity of trigeminal nerve endings and induces more and perhaps larger nerve impulses to transmit signals to the CNS, which in turn lowers the IOP (Ling et al., 2016). Thus, it is evident that TRPA1 is a promising target to lower IOP.

Besides that, TRPA1 expression rises when the retina is rendered ischemic, whereas its antagonism can reduce increases in LDH expression levels induced by hypoxia and glucose deprivation (Araujo et al., 2017). These effects suggest that TRPA1 antagonists have a beneficial effect on retinal cell function. Moreover, TRPA1 is capable of working as an oxidative stress receptor, promoting inflammatory reactions and tissue damage by amplifying stress signals (Trevisan et al., 2014). Later, Araújo et al. demonstrated that both pharmacological blockade and genetic deletion of TRPA1 channel in the mouse model of retinal ischemia can attenuate the increased level of caspase-3, reduce retinal cell death and preserve the thickness of retina. The caspase-3 is the marker of apoptosis and oxidative stress. This indicated that TRPA1 could facilitate the retinal damage induced by I/R (de Araujo et al., 2020). These phenomena indicate that TRPA1 may contribute to promoting the process.

### TRP Canonical Channels

#### Expression of TRP Canonical Channels in Ocular Structures Associated With the Pathogenesis of Glaucoma

The TRP canonical (TRPC) subfamily contains seven different subtypes (Clapham, 2003). Initially, Sugawara et al. identified mRNAs and immunofluorescence signals for several transient receptor potential channel homologs (TRPC1, TRPC3, TRPC4, and TRPC6) on the bovine ciliary body with qRT-PCR and fluorescence microscopy, respectively (Sugawara et al., 2006). Then, TRPC1 and TRPC4 channel proteins were found to exist in the TM (Abad et al., 2008). Recently, Oda et al. also identified high levels of TRPC5 expression in mature RGCs (Yamamoto et al., 2020).

#### Function of the TRP Canonical Channels in Glaucoma

The TRPC1 and TRPC4 channels can modulate the function of TM to improve the efficiency of AH outflow. Abad et al. found that TRPC1 and TRPC4 channel proteins along with Ca^2+^ release-activated Ca^2+^ channels (CRACs) are involved in this process by regulating extracellular calcium concentrations, eventually influencing the outflow channel (Abad et al., 2008; Carreon et al., 2017).

In addition, TRPC1 and TRPC6 channels are involved in the development of RGCs and lamina cells. TRPC1 and TRPC6 channels are mechanosensitive ion channels (Maroto et al., 2005; Spassova et al., 2006), and participate in the fibrotic response in other tissues such as the heart, liver, and pancreas (Seth et al., 2009; Wu et al., 2010). The fibrosis reaction in the lamina structure plays an important role in the development of glaucoma (Wallace and O’Brien, 2016). A previous report showed that TRPC6 localizes mainly within the RGC layers in the retina. Its activation can protect RGCs against death in a rat model of retinal ischemia-reperfusion *via* the brain-derived neurotrophic factor (BDNF) pathway (Wang et al., 2010). However, recent research reported elevated levels of TRPC1/TRPC6 channel proteins in glaucoma lamina cells (LC) compared to LC cells from healthy donors. An intervention designed to inhibit TRPC in the LC region contributed to the prevention of progressive LC fibrosis (Irnaten et al., 2020). Moreover, in a randomization study, the concentration of TRPC6 in blood leukocytes of primary open-angle glaucoma (POAG) patients was twice as high as that in healthy people, and the expression level of TRPC6 was correlated with IOP and the cup-to-disk ratio, suggesting that the TRPC6 channel protein may be involved in the development of glaucoma and has the potential to become a biomarker for POAG (Chen et al., 2013). In conclusion, it is reasonable to suppose that the TRPC6 plays a key role in glaucoma.

A previous report showed that the activation of the TRPC5 channel inhibited axonal outgrowth in rat hippocampal neurons (Greka et al., 2003). Additionally, Oda et al. hypothesized that TRPC5 may have similar functions in RGCs. More recently, it was shown that TRPC5 activation might induce axonal remodeling, ultimately leading to cell death (Yamamoto et al., 2020). A substantial body of research shows that the TRPC channels play an important role in glaucoma.

### Transient Receptor Potential Melastatin Receptor Channels

#### Expression of Transient Receptor Potential Melastatin Receptor Channels in Ocular Structures Associated With the Pathogenesis of Glaucoma

There are eight subtypes of the transient receptor potential melastatin receptor (TRPM) family (TRPM1-TRPM8), and the expression of TRPM channels of different subtypes can be observed on the ciliary body and retina (Jimenez et al., 2020). The TRPM3 and TRPM5 channels are currently relevant to glaucoma research, with abundant expression of the *Trpm3* gene observed in the ciliary body (Okumus et al., 2013).

#### Function of the Transient Receptor Potential Melastatin Receptor Channels in Glaucoma

Seydi Okumus et al. conducted a genetic polymorphism survey on the Turkish population to investigate the relationship between TRPM channel gene variation and the risk of POAG for the first time. The study showed that the polymorphism in the TRPM5 gene *rs34551253 (Ala456Thr)* may be associated with an increased risk of POAG in the Turkish population (Okumus et al., 2013). Moreover, the *Trpm3* gene on the ciliary body maintains the continuous inward flow of calcium ions required for ciliary muscle contraction, and the contraction process can control IOP by affecting TM (Hughes et al., 2012). In addition, Bennett et al. found that the *TRPM3* gene on human chromosome 9q is a novel gene for autosomal dominant cataracts and hypertensive glaucoma, which provides new ideas for congenital genetic screening (Bennett et al., 2014).

## Conclusion

Transient receptor ion potential channels are indispensable for maintaining normal ocular function despite exposure to stresses such as increases in mechanical stress, IOP and medium hyperosmolarity that can induce glaucoma symptomology. Based on their role in the process of glaucoma, more research is needed to broaden our understanding on how they modulate the functions that affect glaucoma progression. Furthermore, research is needed to identify small molecules that can be extracted from the plant world to selectively modulate their function. This approach offers the opportunity to isolate substances that have fewer side effects than those caused by drugs that are chemically synthesized.

## Author Contributions

LM and XL conceived and designed the review. LM and HC collected the literature and wrote the manuscript. QL and SJ were involved in the polishing and proofing of the manuscript. HL critically revised the manuscript and provided the funding. All authors have revised the manuscript for important intellectual content and approved the final version to be published.

## Conflict of Interest

The authors declare that the research was conducted in the absence of any commercial or financial relationships that could be construed as a potential conflict of interest.

## Publisher’s Note

All claims expressed in this article are solely those of the authors and do not necessarily represent those of their affiliated organizations, or those of the publisher, the editors and the reviewers. Any product that may be evaluated in this article, or claim that may be made by its manufacturer, is not guaranteed or endorsed by the publisher.
